# Ecological Management of Crown Gall-Affected Soils: Pathogen Reservoirs, Disease-Suppressive Microbiomes, and Helper Consortia

**DOI:** 10.3390/microorganisms14092089

**Published:** 2026-09-18

**Authors:** Yaojie Shi, Yuewei Wu, Chao Li, Yuzhu Song

**Affiliations:** Research Center of Molecular Medicine of Yunnan Province, Faculty of Life Science and Technology, Kunming University of Science and Technology, Kunming 650500, China; 20252218043@stu.kust.edu.cn (Y.S.); 18388961423@163.com (Y.W.)

**Keywords:** crown gall disease, soil microbiome, helper consortia, indigenous antagonists, disease-suppressive soils

## Abstract

Crown gall remains difficult to manage because pathogenic agrobacteria can persist in soil, rhizosphere niches, infected roots, plant residues, and propagative materials, while repeated wounding creates new infection sites. This review examines how these reservoirs interact with soil and plant-associated microbiota to shape disease recurrence. Crown gall-specific studies show that gall and graft-union communities are spatially structured and identify pathogen competition, opine use, and virulence-related functions as processes that may shape disease-associated microbial assemblages. Evidence from better-characterized suppressive soils further identifies niche occupation, resource competition, bacterial–fungal interactions, and community stability as mechanisms worth testing in crown gall systems. Sanitation, wound protection, and single-strain biocontrol remain central to preventive management in nurseries and perennial production systems, whereas microbiome-based approaches remain complementary and experimental. Evaluation of locally adapted microbial consortia should consider pathogen rebound in infection-relevant niches, persistence of protective microorganisms, functional activity, new gall incidence, soil conditions, and non-target effects. Distinguishing crown gall-specific evidence from cross-pathosystem inference will help define testable mechanisms and appropriate criteria for field evaluation.

## 1. Introduction

Soil-borne disease severity depends on more than pathogen abundance. In the rhizosphere, competition for nutrients and colonization sites, microbial antagonism, and recovery after disturbance can determine whether the residual inoculum expands or remains constrained [1,2]. These processes are particularly relevant in perennial and nursery systems, where pathogen reservoirs may persist across production cycles. Crown gall provides a useful example because disease recurrence requires both persistent agrobacteria and repeated access to wounded plant tissues [3].

Crown gall is a persistent soil-borne disease with strong links to soil and rhizosphere ecology. It is caused by pathogenic agrobacteria, particularly tumor-inducing (Ti) plasmid-bearing lineages historically referred to as *Agrobacterium tumefaciens*, and affects fruit trees, ornamental plants, nursery seedlings, and many other horticultural crops [4]. In this review, the term pathogenic agrobacteria refers to Ti plasmid-bearing agrobacterial lineages that cause crown gall disease. Infection usually requires wounds generated during propagation, grafting, transplantation, pruning, mechanical injury, or environmental stress. After pathogenic agrobacteria colonize susceptible wound sites, transfer DNA (T-DNA) from the Ti plasmid is integrated into the plant genome, resulting in stable host transformation and gall formation. Once galls have formed, conventional control measures rarely reverse established symptoms.

The difficulty of managing crown gall extends beyond the visible gall itself. In nurseries and perennial production systems, latent infection, recurrent wounds, contaminated rhizosphere soil, infected roots, plant residues, and propagative materials can sustain pathogen pressure across seasons and production stages. Pathogenic agrobacteria may remain in these reservoirs even after visible galls are removed or local disease pressure is temporarily reduced. Repeated propagation, transplantation, pruning, and environmental stress then create new infection windows. Crown gall management therefore needs to address pathogen entry, early infection, and the soil ecological conditions that allow long-term reservoir persistence.

Conventional measures remain necessary in crown gall management. Clean planting materials, quarantine, sanitation, tool disinfection, wound management, chemical or antibiotic treatments, and single-strain biocontrol can reduce pathogen introduction and early infection risk. Classical antagonistic strains, including *Agrobacterium radiobacter* K84 and the engineered derivative K1026, also show the practical value of biological control under suitable conditions. These measures are mainly preventive, and their efficacy varies with pathogen population, host, soil conditions, and production practices. They can reduce pathogen load or protect wound sites, but they are not designed to restore resident microbial functions associated with durable disease suppression.

Crown gall develops within a series of connected microbial habitats, including soil, the rhizosphere, root surfaces, wound sites, and gall tissues, where resident microorganisms can interact with pathogenic agrobacteria, the plant host, and one another. Available studies indicate that disease status and plant compartment can alter these microbial assemblages, but crown gall-associated microbiomes have been examined in far fewer studies than the microbiomes associated with several major soil-borne diseases. Information is particularly limited for fungal communities, cross-kingdom interactions, microbial network organization, and the functional traits associated with disease suppression. This gap makes it difficult to define the ecological features that distinguish disease-conducive from disease-suppressive soil states in crown gall systems.

Disease recurrence in crown gall-affected soils may reflect pathogen persistence and weak ecological resistance. Indigenous antagonists may already occur in soil and rhizosphere niches, but their abundance, persistence, spatial distribution, or functional expression can be constrained by site-specific pH, nutrient availability, organic-matter status, moisture conditions, community instability, or poor colonization of infection-relevant niches. Under disease-conducive conditions, indigenous protective guilds may not adequately restrict pathogenic agrobacteria. In crown gall-affected soils, disease suppression may depend on resident microbial functions such as antagonism, occupation of infection-relevant niches, community resilience, and recovery after disturbance.

Locally adapted antagonists may also benefit from microbial partners that improve their performance in soil. These partners need not inhibit pathogenic agrobacteria directly. Some may support colonization or biofilm formation; others may improve resource acquisition or help protective populations remain active under environmental stress. We use the term helper consortium for combinations in which partner microorganisms improve the establishment or function of a core antagonist. Helper consortia are considered here as an extension of ecological management, not as a replacement for sanitation, wound protection, or established biological control.

Three questions organize this review: where pathogenic agrobacteria persist between infection events, which microbial processes may restrict their recovery in crown gall-affected soils, and how locally adapted microbial combinations can be evaluated given the current evidence. These questions are considered through pathogen reservoirs, existing control measures, soil suppressiveness, consortium design, and field implementation.

## 2. Reservoir Ecology of Crown Gall Disease

### 2.1. T-DNA-Mediated Host Transformation and the Limited Window for Curative Control

The pathogenesis of crown gall leaves only a narrow window for intervention before stable host transformation occurs. Pathogenic agrobacteria carry Ti plasmids that encode the T-DNA region and the virulence machinery required for host transformation [5]. After activation by wound-derived plant signals, the *vir* system mediates T-DNA processing and transfer into host cells, followed by integration into the plant genome [6,7,8]. This process genetically reprograms infected plant tissues, alters hormone regulation, and induces uncontrolled cell proliferation and gall formation [4,9]. After T-DNA integration and tumor development, reducing bacterial abundance may lower secondary inoculum pressure, but established gall symptoms are unlikely to be reversed [10].

Because stable host transformation is difficult to reverse, management is most likely to succeed before T-DNA transfer and tumor establishment. Reducing the reservoir, protecting fresh wounds, and limiting bacterial establishment at the root and wound surfaces are therefore more realistic targets than attempting to reverse an established gall. Recurrent crown gall reflects repeated contact between persistent inoculum and susceptible plant tissues; detection of the pathogen alone does not fully describe disease risk.

T-DNA-mediated opine synthesis also links molecular pathogenesis with soil and rhizosphere ecology. Opines produced in transformed tissues create specialized nutrient niches for opine-utilizing bacteria [11]. Because opine catabolism is not restricted to pathogenic agrobacteria, these metabolites may shape the wider gall-associated community while also supporting the local persistence of pathogenic populations. Gall tissues may therefore provide selective microbial habitats shaped by tumor-derived resources and local bacterial interactions. The irreversibility of host transformation and the formation of selective gall-associated nutrient niches together limit purely curative control strategies.

### 2.2. Soil and Propagative Materials as Long-Term Pathogen Reservoirs

Pathogenic agrobacteria can persist in soil, rhizosphere environments, infected roots, plant residues, and propagative materials [3,12]. These reservoirs maintain disease pressure across production cycles, particularly in nurseries and perennial cropping systems. Infected seedlings, contaminated rhizosphere soil, root fragments, and planting materials can disseminate pathogenic agrobacteria from localized infection sites to new production areas [13]. Even after visible galls are removed or local infection is temporarily suppressed, residual pathogenic populations may remain in soil and plant-associated niches.

These reservoirs are epidemiologically important because they can repeatedly supply inoculum to root surfaces and newly formed wounds. Crown gall-affected soils can support pathogenic agrobacteria for long periods when soil conditions and resident microbial communities provide insufficient ecological resistance [12]. Long-term management therefore needs to lower reservoir pressure in soil, the rhizosphere, root-associated tissues, and propagation-related materials. Removal of visible galls alone cannot address these reservoirs.

Residual inoculum also contributes to the inconsistent field performance of short-term treatments. A local reduction in bacterial abundance does not remove residual populations from soil, roots, or propagative material, and these residual populations can recolonize roots or fresh wounds when new infection sites become available. The persistence and spatial distribution of inoculum therefore need to be considered alongside the immediate effect of a treatment.

### 2.3. Rhizosphere and Wound Niches as Recurring Infection Interfaces

Rhizosphere and wound-associated niches are where soil reservoirs most directly contribute to new infections. Crown gall infection usually requires wounds generated during propagation, grafting, transplantation, pruning, mechanical injury, freezing, drought, or other environmental stresses. These wounds provide entry sites for pathogenic agrobacteria and release plant-derived signals that activate infection-related bacterial functions [8]. Routine production practices and environmental disturbances can therefore generate repeated infection windows even under moderate pathogen pressure.

The rhizosphere further shapes disease risk by regulating microbial movement, resource availability, pathogen–host contact, and competition among resident microorganisms [14]. In disease-conducive soils, pathogenic agrobacteria may occupy rhizosphere and wound niches before protective microorganisms become established. Early pathogen occupation increases the probability of wound colonization, T-DNA transfer, and gall initiation. In contrast, the rapid establishment of protective microorganisms in these niches may reduce pathogen access to susceptible tissues and interfere with pathogen growth, signaling, or early colonization.

Pathogen abundance measured in bulk soil provides only a partial indication of suppressive potential. The timing, location, and functional activity of microorganisms in rhizosphere and wound-associated niches are also critical. A soil may contain potentially antagonistic microorganisms and still remain disease-conducive if those organisms are absent from infection sites or inactive when wounds become available. For crown gall, the protective effect of a microbial population depends not only on its abundance, but also on whether it is active at the relevant site and time.

### 2.4. Crown Gall-Associated Microbial Communities and Ecological Niches

The microbial environment associated with crown gall is spatially heterogeneous. Bulk soil, rhizosphere soil, root surfaces, wound sites, and gall tissues represent distinct habitats, and the microbial community detected in one compartment cannot be assumed to represent another. Gall formation further changes plant metabolism and local nutrient availability, creating a niche that differs from healthy root or stem tissues. Consequently, crown gall-associated microbiota should be examined at the compartment level rather than treated as a single community surrounding the pathogen.

Direct evidence is available mainly from grapevine crown gall systems. For taxonomic consistency, *Allorhizobium vitis* is used throughout this review for the taxon historically referred to as *Agrobacterium vitis* or *Rhizobium vitis*; the recently proposed combination *Gillisella vitis* is noted where relevant [15]. Faist et al. compared soil, roots, graft unions, and canes from diseased and non-diseased grapevines over a growing season and found that plant compartment explained more variation in bacterial community composition than disease status itself [16]. Crown gall disease had its clearest effect at the graft union. Unlike healthy graft unions, whose dominant bacterial members changed across seasons, gall-bearing graft unions contained a more stable community repeatedly dominated by *A. vitis*, *Pseudomonas* spp., and members of Enterobacteriaceae [16]. Gall-bearing tissues therefore represent a distinct microbial habitat, but the disease-associated signal remains strongly compartment dependent.

Gall-associated communities also vary geographically. A survey of 73 grapevine crown galls collected from vineyards in Hungary, Japan, Tunisia, and the United States found marked differences in gall-associated bacterial communities among sampling sites [17]. *A. vitis* was frequently accompanied by taxa assigned to *Xanthomonas, Novosphingobium*, Microbacteriaceae, and other bacterial groups, although these co-occurrence patterns do not establish whether the associated organisms promote or restrict disease. No fixed crown gall microbiome has emerged across the available studies. Gall tissue appears to provide a selective habitat, but the community that develops within it remains strongly dependent on host, site, season, and the local microbial pool. The small number of crown gall microbiome studies is still insufficient to identify taxa or interactions that consistently accompany disease suppression.

### 2.5. Disease-Conducive Soil States and Insufficient Ecological Resistance

Persistence of pathogenic agrobacteria in crown gall-affected soils reflects both pathogen survival and limited ecological resistance from indigenous microbial communities. Protective microorganisms may be present but contribute little to disease suppression when they are sparse, poorly established at the roots or wound-associated niches, or functionally inactive under local soil conditions. Disease risk therefore depends on the ecological setting in which residual inoculum persists, not simply on pathogen presence.

**Figure 1:**
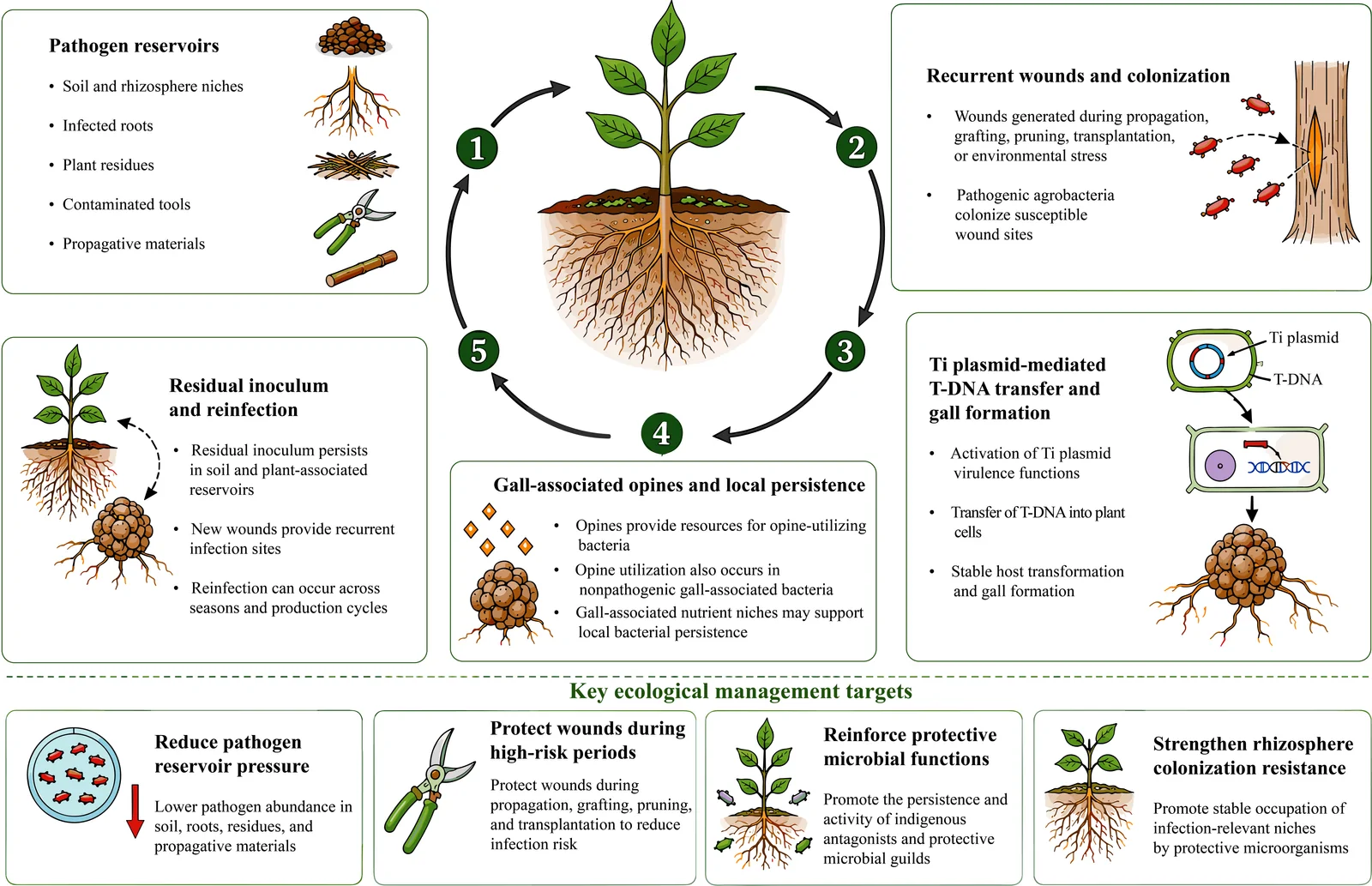
Reservoir-driven persistence and recurrence of crown gall in soil and plant-associated habitats. The numbered sequence represents (1) pathogen reservoirs, (2) recurrent wounding and wound colonization, (3) Ti plasmid-mediated T-DNA transfer and gall formation, (4) gall-associated opine use and local microbial persistence, and (5) residual inoculum and reinfection. Pathogenic agrobacteria can persist in soil and rhizosphere niches, infected roots, plant residues, contaminated tools, and propagative materials. Recurrent wounding creates susceptible infection sites, followed by T-DNA transfer, stable host transformation, and gall formation. Opine-rich gall-associated niches may support both pathogenic and nonpathogenic opine-utilizing bacteria, while residual inoculum can contribute to subsequent infection cycles. The unnumbered lower panel summarizes four ecological management targets: reducing pathogen reservoir pressure, protecting susceptible wounds during high-risk periods, reinforcing protective microbial functions, and strengthening rhizosphere colonization resistance.

For crown gall, suppressiveness is better defined by pathogen behavior and protective microbial function than by pathogen absence. Pathogenic agrobacteria may remain detectable, but a suppressive state would be characterized by restricted pathogen growth, reduced access to susceptible tissues, weaker rebound after disturbance, and persistent protective microbial functions. Candidate mechanisms underlying this state are examined in Section 4, and Figure 1 summarizes the reservoir-driven cycle linking pathogen persistence, recurrent wounding, and renewed infection.

## 3. Existing and Emerging Crown Gall Control Strategies and Their Ecological Boundaries

### 3.1. Agronomic and Preventive Management

Agronomic and preventive measures remain the first line of crown gall risk reduction. Clean planting materials, nursery quarantine, sanitation, tool disinfection, wound reduction, stress mitigation, and careful handling during grafting, pruning, and transplantation can limit pathogen introduction and reduce wound-associated infection opportunities. Pathogenic agrobacteria can be disseminated with infected seedlings, contaminated rhizosphere soil, root fragments, and propagative materials, making early exclusion and sanitation especially important [13].

Once pathogen reservoirs are established, preventive measures become less effective. Latent infection can be difficult to detect, and nurseries or open-field systems continue to experience soil movement, irrigation, transplantation, pruning, and environmental injury. These conditions make the complete exclusion of pathogenic agrobacteria unrealistic over long production cycles. Preventive measures remain essential for reducing new introductions and limiting infection opportunities, but they have little direct effect on the resident microbial processes that determine whether an established soil remains disease-conducive [12,13].

### 3.2. Chemical, Antibiotic, and Wound-Protection Approaches

Chemical, antibiotic, and wound-protection approaches are mainly used to lower local pathogen load and protect vulnerable wound sites. They are most relevant during grafting, pruning, transplantation, and other high-risk operations in which fresh wounds provide entry points for pathogenic agrobacteria. Their effects are largely confined to early disease development. Although these approaches can reduce early infection pressure, their value against established galls is limited after T-DNA-mediated host transformation has occurred [18].

Repeated broad-spectrum antimicrobial use can disrupt non-target microbial communities, alter community composition, and weaken protective microbial functions. Antibiotic use raises additional concerns about resistance selection and the dissemination of resistance determinants through horizontal gene transfer. Their principal value is short-term pathogen suppression or wound protection, whereas durable modification of the resident soil microbiome is unlikely to result from these treatments alone.

### 3.3. Single-Strain Biocontrol: Achievements and Ecological Constraints

Single-strain biocontrol is one of the most established biological approaches for crown gall management. Representative examples include *A. radiobacter* K84 and its engineered derivative K1026. K84-type biocontrol is mainly associated with agrocin-mediated inhibition of susceptible pathogenic agrobacteria and has practical value in nursery and wound-protection contexts [19,20]. K1026 was developed to reduce risks associated with transfer of the agrocin-associated plasmid [21,22]. These strains demonstrate the practical value of targeted biological control when the pathogen is susceptible and the antagonist reaches the relevant infection niche.

K84 and K1026 also illustrate the ecological limits of single-strain biocontrol. Crown gall recurrence is shaped by soil reservoir persistence, rhizosphere and wound colonization, wound-signal activation, T-DNA transfer, host transformation, and resident microbial community structure. A single antagonistic strain or metabolite is unlikely to suppress the pathogen consistently across different niches, seasons, and production stages. Field performance can also be influenced by soil physicochemical properties, organic matter, moisture, nutrient availability, temperature, resident microbiota, pathogen population structure, and seasonal variation.

Many of the ecological limitations become apparent only after an antagonist is introduced into non-sterile soil. Its performance depends on whether the strain can occupy the required niche before the pathogen, use locally available resources, tolerate soil stress, and remain active in the presence of resident competitors and predators [23,24]. Priority effects can be important at wound and root surfaces because early occupation may determine which population gains access to plant-derived nutrients [25]. Colonization resistance from the resident microbiota can also restrict an introduced biocontrol strain, even when the same strain performs strongly in agar or liquid assays.

Functional expression is another source of inconsistency. Production of antimicrobial metabolites, siderophores, extracellular matrix, or signaling-interference molecules can change with nutrient availability, pH, oxygen status, population density, and interaction with neighboring microorganisms. Thus, failure in the field does not necessarily mean that a strain lacks antagonistic capacity. The required function may simply not be expressed at the right place or time. Strong in vitro inhibition alone is insufficient for selecting field inoculants.

Single-strain biocontrol is most effective when an antagonist reaches the relevant infection niche and the target pathogen is susceptible. Microbiome-oriented approaches address a different problem: the persistence of protective functions and community-level resistance beyond the immediate infection site [23,24,25].

### 3.4. Emerging Biological and Microbiome-Oriented Strategies

More recent biological approaches have broadened crown gall control beyond the classical K84/K1026 system [26]. Nonpathogenic *A. vitis* strains, including ARK-1, have been examined for the suppression of grapevine crown gall, including effects on pathogen colonization and virulence-related processes [27,28]. *Bacillus velezensis* strains have also received increasing attention because they combine direct antimicrobial activity [29] with biofilm formation and plant defense induction [30,31,32]. Their field performance, however, still depends on successful colonization, local soil conditions, and the pathogen population present [33].

Host genotype and root chemistry can also alter crown gall risk. Resistant rootstocks and altered root exudation can change pathogen recruitment before infection and may also influence rhizosphere community assembly [34]. Work in *Prunus* and *Rosa* systems indicates that changes in root-derived metabolites can influence colonization by pathogenic agrobacteria [35].

Microbiome-oriented approaches remain much less developed for crown gall. Organic amendments, native microbiome enrichment, suppressive-soil management, and synthetic communities have been explored more extensively in other soil-borne disease systems. Their relevance to crown gall lies in the possibility of modifying community-level functions that a single antagonist cannot maintain on its own. At present, these approaches should be viewed as experimental directions rather than established crown gall control measures.

### 3.5. Why Short-Term Pathogen Suppression Does Not Ensure Durable Disease Control

Short-term reductions in pathogen abundance or wound infection do not necessarily indicate durable disease suppression. Residual agrobacterial populations can persist in soil and plant-associated niches and recover when new infection sites become available. Durable suppression would be expected to involve the sustained restriction of pathogen rebound together with protective microbial activity in infection-relevant niches. The ecological processes underlying this distinction are examined in Section 4, while the scope and limitations of the current management approaches are summarized in Table 1.

## 4. Ecological Processes Shaping Disease Conduciveness and Suppressiveness

### 4.1. From Disease-Conducive to Disease-Suppressive Soil States

Disease suppressiveness is not equivalent to the absence of a pathogen. In many soil-borne disease systems, the pathogen remains detectable while disease stays low because the surrounding community restricts growth, access to resources, or colonization of the host [2,51,52]. Persistent agrobacteria can remain in soil or plant-associated reservoirs for long periods, yet infection still requires successful access to newly wounded tissue.

In better-characterized suppressive soils, disease reduction generally reflects a combination of broad community-level resistance and more specific antagonistic activities. Community-level resistance can restrict pathogen expansion through resource competition and niche occupation, whereas specific populations or functions may interfere directly with colonization, signaling, or virulence [53,54,55,56]. Both processes are plausible in crown gall, although their relative contributions have not been resolved.

Crown gall-suppressive soils have not been characterized as extensively as classical suppressive systems such as take-all or Fusarium wilt. The following sections distinguish three levels of evidence: mechanisms demonstrated directly in crown gall systems, mechanisms supported by other soil-borne disease systems but not yet verified in crown gall, and hypotheses proposed for future testing. Evidence from related pathosystems is used to generate testable hypotheses rather than to imply that the same mechanisms have already been demonstrated in crown gall. These evidence levels are summarized in Table S1, which also identifies the evidence basis and the principal limitation or next test for each mechanism or management concept.

### 4.2. Bacterial, Fungal, and Cross-Kingdom Interactions

Fungal contributions remain poorly characterized in crown gall microbiome research. Most studies have focused on bacteria, reflecting both the bacterial etiology of crown gall and the long history of bacterial biocontrol with K84, K1026, ARK-1, and *Bacillus* spp. As a result, it remains unclear whether bacterial profiles alone capture the ecological differences between disease-conducive and low-disease soils.

Studies of other soil-borne diseases indicate several mechanisms by which fungi may contribute to disease suppression. Some fungal groups contribute directly through competition or antagonistic metabolites; others alter decomposition, nutrient turnover, root physiology, or the physical structure of the rhizosphere. Their influence may therefore be indirect, acting through bacterial recruitment or resource availability rather than through direct inhibition of the pathogen [57]. In a related soil-borne disease system, *Mortierella* has been associated with stronger suppressive activity of resident microbiota [58]. Cross-kingdom synthetic communities also show that bacterial and fungal members can contribute different functions within the same rhizosphere [59,60,61].

Direct crown gall evidence is still insufficient to identify fungal taxa that consistently associate with low disease or alter pathogen colonization. Inclusion of a fungal member would therefore be justified only when a measurable contribution, such as improved root association, resource turnover, stress tolerance, or antagonist persistence, can be demonstrated. Cross-kingdom composition alone does not demonstrate suppressive value. Evidence from other host-associated systems further indicates that resident symbionts can alter pathogen colonization through resource competition, direct inhibition or facilitation, and host-mediated effects, although these mechanisms require independent validation in crown gall [62].

### 4.3. Microbial Networks, Keystone Taxa, and Community Stability

Taxonomic composition gives only a partial view of the crown gall-associated community. The same taxa can occur in different soils or galls while their associations with one another differ markedly. Co-occurrence analysis of grapevine crown galls has identified recurrent associations between *A. vitis* and members of Rhizobiaceae, *Xanthomonas*, *Novosphingobium*, Methylocystaceae, and Microbacteriaceae [17]. The functional meaning of these associations remains unresolved, but the recurrent co-occurrence patterns indicate that the pathogen occurs within a structured microbial assemblage rather than as an isolated population.

Dominant taxa should not be equated with keystone taxa. High abundance may simply reflect adaptation to the gall environment. A less abundant organism can be more influential if it supports several community members or occupies a position that affects community stability [63]. Studies of suppressive soils in other pathosystems have moved beyond correlation by isolating candidate taxa identified through network analysis and testing their effects on the pathogen and disease. Network position should therefore be used to nominate candidate taxa for experimental testing, not as evidence that a taxon drives disease suppression. For crown gall, taxa that recur across low-disease sites, hosts, or seasons would be higher-priority candidates for isolation and subsequent community experiments. Comparable analyses in other chronic polymicrobial diseases have shown that microbial composition, niche preference, and interaction networks can shift across disease-severity gradients, underscoring the value of compartment-resolved sampling when candidate keystone taxa are identified [64].

### 4.4. Functional Genes, Pathways, and Metabolites

Functional ecology is particularly relevant to crown gall because pathogen abundance alone does not indicate infection competence. Successful infection depends on wound sensing, chemotaxis, attachment, Ti plasmid-associated virulence regulation, and T-DNA transfer. Biological control can act on these functions without eliminating the pathogen. For example, nonpathogenic *A. vitis* strain ARK-1 suppresses the expression of virulence-related genes in tumorigenic strains of the same species, illustrating how infection potential can be reduced without eliminating the pathogen [28].

Interbacterial competition provides another direct link between functional genes and the crown gall microbiome. Agrobacterial type VI secretion system (T6SS) mutants showed lower disease incidence than the wild type in a soil-inoculation model, and T6SS activity also altered the gall-associated microbiome (gallobiome) [65]. The effect depended on season, and the pathogen used T6SS-mediated competition against a rhizosphere *Sphingomonas* isolate [65]. Thus, a bacterial competition system can influence both infection success and the microbial community encountered during disease development.

Gall-associated resource use is also functionally important. Opine-catabolism genes occur not only in virulent agrobacteria, but also in several nonvirulent bacteria isolated from grapevine crown galls, suggesting that tumor-derived metabolites can select for a broader group of gall residents [66]. Opines may therefore act as community-level resource filters rather than serving only as substrates for pathogenic agrobacteria.

Crown gall biocontrol studies have identified antimicrobial lipopeptides [36,67,68], macrolactin-associated activity [37], agrocin-mediated inhibition, biofilm-related traits, and plant defense induction. In other disease-suppressive rhizospheres, nonribosomal peptide synthetase-associated functions have also been linked to suppression [56,69]. Suppressive potential cannot be inferred from taxonomic abundance alone and instead depends on whether competitive, antimicrobial, colonization, or anti-virulence functions are expressed at infection-relevant sites.

Metagenomic screening can identify candidate functional genes, but mechanisms that depend on environmental conditions require evidence of activity rather than gene presence alone. Metatranscriptomics, targeted expression assays, or metabolomics can then be used to test whether the relevant pathways are active under disease-suppressive conditions. Integrated microbiome and metabolome analyses in other disease-associated systems provide a methodological precedent for linking predicted microbial potential with condition-specific metabolic activity [70].

### 4.5. Soil Properties and Management as Ecological Filters

Soil management can influence disease by changing the physicochemical environment in which microbial communities assemble and function. pH, moisture, organic matter, nutrient availability, aeration, and soil structure do not act independently of the microbiome [1,71]. They determine which organisms can establish, how rapidly resources are consumed, and whether protective populations remain active after disturbance. The same pathogen population can therefore encounter very different levels of ecological resistance in different soils.

The effects of organic amendments are strongly context dependent. An amendment can increase carbon supply, improve aggregation, and stimulate resident microorganisms, creating conditions that favor competitive or antagonistic populations [38,39]. The same addition can also increase readily available substrates for pathogenic agrobacteria if protective guilds are weak. More microbial biomass is therefore not necessarily equivalent to greater suppressiveness. The value of an amendment depends on which organisms and functions it favors.

pH adjustment, irrigation, and nutrient management can likewise reshape the microbial environment in which pathogens and protective populations compete. Adjusting pH changes nutrient and metal availability and can shift the relative fitness of bacterial and fungal groups. Irrigation influences bacterial movement and root stress as well as microbial activity. Excess nutrient availability may favor rapid pathogen recovery, whereas avoiding large pulses of readily available nutrients may limit rapid pathogen regrowth and support a more stable rhizosphere community. These responses are likely to vary among soil types and production systems.

Management also acts through the plant. Root exudation changes with genotype, rootstock, nutrient status, and stress [72,73], altering the chemical environment in which rhizosphere organisms are recruited [74,75,76]. For crown gall, this matters because root-derived compounds influence agrobacterial colonization. Reduced soil disturbance may further preserve the spatial organization of resident microbial communities, while repeated chemical or physical disturbance may reduce sensitive protective populations or disrupt their spatial organization. Soil management and inoculant performance should therefore be evaluated together [40,77].

### 4.6. Integrating the Drivers of Soil Conduciveness and Suppressiveness

**Figure 2:**
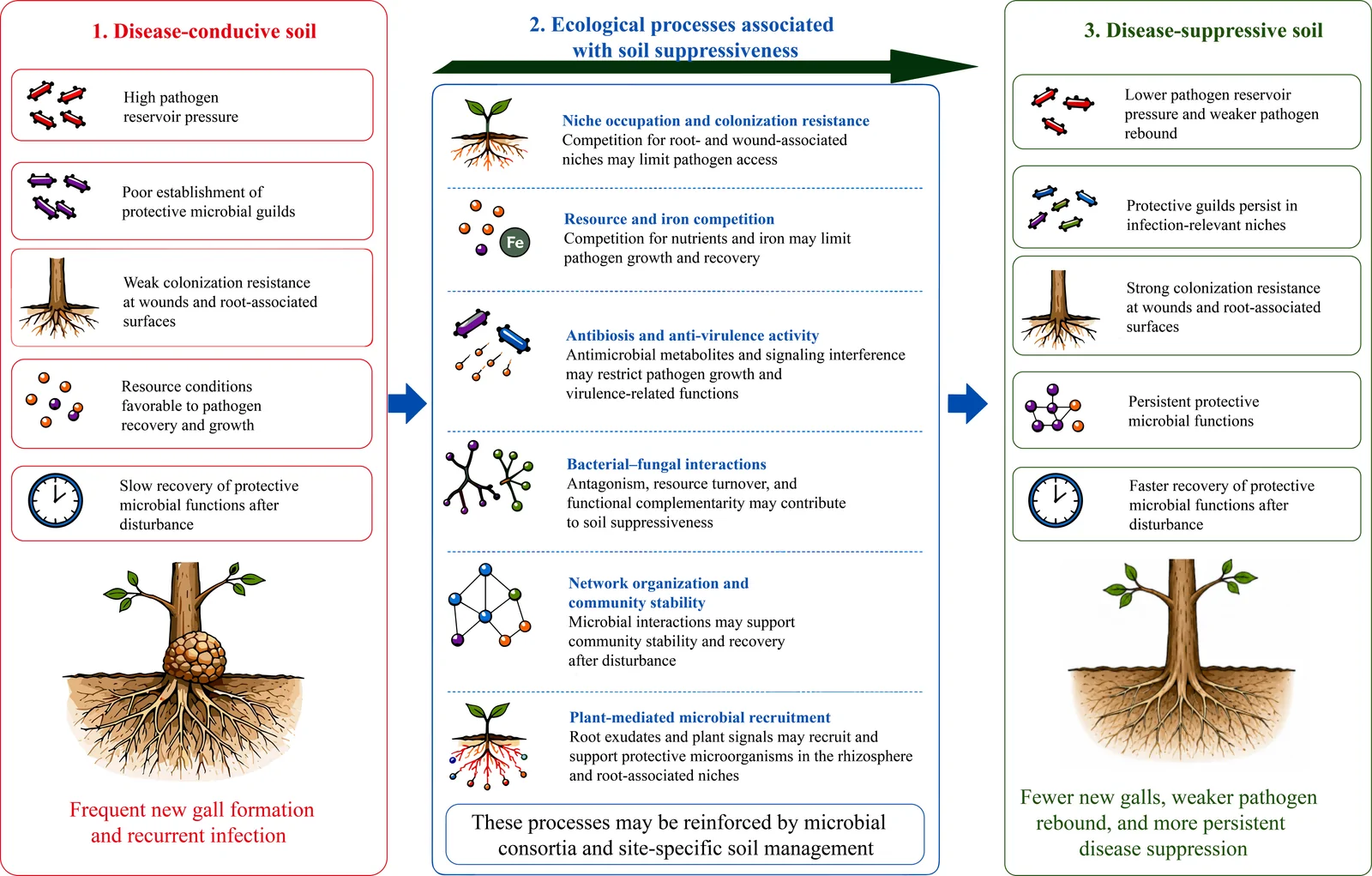
Ecological processes associated with the transition from disease-conducive to disease-suppressive soil states in crown gall systems. The three panels represent (1) a disease-conducive state characterized by high pathogen reservoir pressure and weak colonization resistance, (2) candidate ecological processes associated with soil suppressiveness, and (3) a disease-suppressive state characterized by weaker pathogen rebound and persistent protective microbial functions. Candidate processes include niche occupation and colonization resistance, competition for nutrients and iron, antibiosis and anti-virulence activity, bacterial–fungal interactions, network organization and community stability, and plant-mediated microbial recruitment. These processes may be reinforced by microbial consortia and site-specific soil management. The lower statements correspond to the expected disease outcomes of the disease-conducive state on the left and the disease-suppressive state on the right, respectively.

Crown gall is most likely to recur when persistent inoculum coincides with accessible wounds, weak colonization resistance, favorable resources, and slow recovery of protective microbiota after disturbance. In a suppressive soil, residual agrobacteria may still be detectable, but their expansion, access to infection sites, and rebound are constrained. Competition, antagonism, interference with virulence, plant-mediated recruitment, microbial interactions, and soil conditions are all plausible contributors, although their relative importance probably differs among soils, hosts, and seasons [78]. Figure 2 summarizes these candidate processes.

## 5. A Proposed Framework for Designing and Evaluating Indigenous Helper Consortia

### 5.1. Defining Core Antagonists and Helper Functions

Core antagonists refer to locally adapted microorganisms that directly restrict pathogenic agrobacteria or interfere with early infection. Their activity may involve antimicrobial metabolites, competition for nutrients or iron, interference with pathogen signaling, occupation of root or wound surfaces, or the induction of plant defense. Several *B. velezensis* strains and nonpathogenic agrobacteria illustrate these forms of pathogen suppression in crown gall systems [28,29,31,36].

Here, helper microorganisms are defined as partners selected for their ability to improve the persistence or activity of a core antagonist or resident protective population. Their contribution may involve improved persistence under unfavorable environmental conditions, resource acquisition, attachment or biofilm formation, or recovery after stress. Strong inhibition of pathogenic agrobacteria in vitro is therefore not a requirement for every candidate. A microorganism with little direct antagonistic activity may still be useful if it reproducibly improves the establishment or function of the core antagonist under relevant soil or plant-associated conditions.

The distinction between core antagonists and helpers is functional rather than taxonomic [43,44]. A microorganism may act mainly as an antagonist in one community and as a facilitator in another, depending on the receiving soil, neighboring organisms, and available resources. Candidate pools can therefore include bacterial or fungal members when they provide a defined ecological function, but taxonomic diversity itself should not be used as a criterion for consortium complexity.

### 5.2. Diagnosing Crown Gall-Affected Soils and Identifying Local Microbial Candidates

Sites are unlikely to share the same limitation to biological suppression. Some may carry a high pathogen reservoir, whereas others may contain moderate pathogen populations but little microbial competition around roots or wounds. In other soils, potentially useful antagonists may already be present but remain poorly active because of pH, moisture, nutrient status, or organic matter. A single consortium or formulation is therefore unlikely to perform equally well across these settings.

Baseline characterization should include pathogen abundance, soil pH, organic matter, nutrient status, moisture, texture, and microbial composition. Because bulk soil may not represent the niches where infection occurs, rhizosphere, root-surface, and wound-associated samples should be included when feasible. Community profiling can identify differences among diseased, low-disease, and healthy sites, while functional screening can determine whether candidate protective traits occur in the local microbial pool. Where sample size permits, network analysis may help identify hub or connector taxa associated with low-disease soils, but their roles require experimental verification [79].

Microbial isolation can then be directed toward habitats where disease remains low despite exposure to the local pathogen pool. Healthy plants within affected orchards, low-disease patches, symptomless rhizospheres, root surfaces, and wound-associated niches are particularly informative because microorganisms recovered from these habitats have already experienced the local soil and host environment. Crown gall-affected soils should not be excluded from sampling, as they may also contain antagonists or helper organisms whose abundance or activity is insufficient for effective suppression. Comparative sampling across these habitats is preferable to screening a large collection of unrelated isolates.

Direct inhibition assays remain useful for identifying core antagonists but are insufficient as the sole screening criterion for helper microorganisms. A candidate that produces no visible inhibition zone may still enhance antagonist growth, improve biofilm formation, increase survival under drought or pH stress, or alter resource availability in a way that disadvantages the pathogen. Candidates should not be excluded solely because of weak direct antibiosis if they reproducibly improve antagonist performance under relevant conditions.

### 5.3. Selecting Compatible Core Antagonists and Helper Microorganisms

Core-antagonist selection begins with reproducible activity against pathogenic agrobacteria under conditions that approximate the intended application. Inhibition strength remains useful, but it should be considered together with rhizosphere or wound colonization, persistence in non-sterile soil, stability across relevant pH and moisture ranges, and compatibility with the host plant [80]. A strain that performs well only on nutrient-rich agar is a poor candidate for field use if the same activity is lost in soil or at the infection site.

Helper candidates require a different screening strategy. Pairwise or small-group assays can test whether a candidate improves the growth, survival, colonization, or functional expression of the selected core antagonist. Depending on the proposed helper function, this may involve co-culture under nutrient limitation, biofilm formation, stress-tolerance assays, resource competition, root colonization, or non-sterile soil microcosms. The relevant endpoint is the change in antagonist performance caused by the helper, not the helper’s individual inhibition of the pathogen.

The absence of inhibition between consortium members is not sufficient to establish compatibility. Two strains may grow together on agar but still compete strongly for the same limiting resource in soil, while strains showing modest growth interference in vitro may occupy different niches and coexist successfully around roots [25]. Compatibility therefore needs to be checked under soil or root-associated conditions and should include spatial establishment, resource use, and maintenance of the intended function.

Fungal candidates can be evaluated using the same functional principle. A fungal member is worth retaining when it improves a defined property of the consortium, such as root association, stress tolerance, or persistence of the core antagonist. Cross-kingdom composition by itself is not a sufficient reason to increase consortium complexity. Microbial functions that may support antagonist performance and their expected ecological outcomes are summarized in Table 2.

### 5.4. Constructing Minimal Functionally Defined Consortia

The smallest consortium that retains the required functions is generally preferable to a larger mixture with poorly defined interactions. Adding strains increases the number of possible interactions, complicates formulation, and makes failure more difficult to interpret. Consortium size should therefore be determined by functional need [45,46]. An additional consortium member is justified when it contributes a function that is absent or unstable in the existing combination, not simply because it performs well as an individual isolate.

Consortium construction can proceed stepwise [47]. A core antagonist is first tested alone, followed by antagonist–helper pairs and then by small combinations in which each added member has a defined role. At each step, the combination should be compared with the best individual strain. A combination that provides neither stronger suppression nor greater stability than the best individual strain does not justify the added complexity.

Studies in other soil-borne disease systems show that minimal and cross-kingdom synthetic communities can reshape rhizosphere communities and suppress disease when the retained members occupy complementary niches [81,82]. For crown gall, a minimal consortium should retain only the members needed to provide stronger or more stable suppression than the core antagonist alone under increasingly realistic conditions.

Validation can proceed from simplified systems to increasingly field-relevant conditions [48,83]. Simplified or sterile systems are useful for identifying direct interactions among consortium members, whereas non-sterile soil tests whether those interactions persist in the presence of resident competitors [84]. Root and rhizosphere systems then add host-derived resources and spatial constraints. Greenhouse experiments connect these ecological processes with disease outcome, while field trials test whether the same functions survive environmental and management variability [48,85].

### 5.5. Criteria for Evaluating Microbiome Rehabilitation

Microbiome rehabilitation refers operationally to a sustained state characterized by weaker pathogen rebound, persistence of protective microbial functions, and the reduced formation of new galls; it does not imply restoration of a presumed pre-disease microbial community. Inoculant survival alone is insufficient evidence of such a shift. Stronger evidence would require persistent disease suppression together with lower pathogen pressure and maintenance of these functions across repeated observations. Persistence of an introduced consortium member without a disease response, or a transient decline in pathogen abundance followed by rebound, would indicate incomplete ecological change.

Disease outcome remains the clearest biological endpoint. For crown gall, the relevant response is the incidence or severity of newly formed galls because existing tumors are not expected to disappear when the soil microbiome changes. Measurements need to extend beyond the first post-treatment period. Delayed recurrence after an initially effective treatment would argue against a durable suppressive state.

Pathogen pressure should be followed over time rather than assessed at a single endpoint. Evidence of a durable response would include a sustained decline in pathogenic agrobacteria within infection-relevant niches and weaker rebound after disturbance or a new production cycle. A decline in bulk soil is less informative when pathogen populations remain high around roots or wound-prone tissues.

Microbial establishment is better demonstrated by the persistence of protective populations and their functions at root surfaces, in the rhizosphere, or near wound sites than by bulk-soil detection alone. Changes in bacterial and fungal community structure can indicate whether the receiving microbiome has shifted, but greater diversity or network complexity should not automatically be interpreted as recovery [86].

Functional measurements are needed to connect community change with the mechanisms underlying disease suppression. Antimicrobial activity, resource competition, biofilm formation, stress tolerance, anti-virulence functions, or related genes and metabolites are most informative when they change in parallel with disease suppression. A taxonomic shift without a corresponding functional or disease response should be interpreted cautiously.

No single indicator is likely to define rehabilitation across all crown gall-affected soils. Microbiome rehabilitation is most convincingly supported when several responses occur together: fewer new galls, sustained reduction in pathogen pressure, weaker pathogen rebound, persistence of protective microorganisms at infection-relevant sites, sustained suppressive activity, and limited disturbance of non-target soil functions. Improvement confined to one level is better described as a partial response than as microbiome rehabilitation. These criteria, together with the relevant sampling niches and monitoring windows, are summarized in Table 3.

### 5.6. From Experimental Validation to Field Deployment

A consortium should advance to field testing only after it shows a reproducible advantage over the core antagonist alone under non-sterile soil and plant-associated conditions [49,84]. This advantage may appear as stronger disease suppression, more stable colonization, greater persistence, or better recovery after environmental stress, but it should remain detectable in the presence of the resident microbiota.

Greenhouse experiments should retain comparisons among the core antagonist, the complete consortium, and untreated controls, with an established biological control treatment included where appropriate. Progression to field trials then requires a suitable formulation, niche-matched delivery, compatibility with production practices, biosafety assessment, and a plan for long-term monitoring.

## 6. Field Implementation, Constraints, Biosafety, and Adaptive Soil Management

### 6.1. Delivery Strategies for Crown Gall-Affected Soils

In field trials, consortium performance will depend strongly on whether viable cells reach the sites where pathogenic agrobacteria persist and infection begins. Relevant targets include the rhizosphere, root surfaces, wound sites, and propagative materials. Root dipping, seedling treatment, wound application, rhizosphere inoculation, and carrier-based soil application target different ecological niches and should not be treated as interchangeable delivery routes [13,23,50,80].

In nursery and perennial systems, delivery timing is as important as consortium composition. Applications before grafting, transplantation, pruning, or anticipated environmental stress may promote the early occupation of rhizosphere and wound-associated niches by protective microorganisms before pathogenic agrobacteria become established. In established crown gall-affected soils, repeated applications and compatible soil management practices may be needed when a single inoculation does not provide sufficient persistence. The selected delivery method needs to match the target niche and field context, including soil properties, cropping system, propagation practices, disease pressure, and management objective. Wound protection requires the rapid occupation of a short-lived infection site, whereas soil-oriented treatment requires longer persistence and interaction with the resident community. These are different delivery problems and should not be evaluated by the same short-term endpoint.

### 6.2. Formulation Stability and Ecological Establishment

Strong in vitro performance does not guarantee soil performance if consortium members cannot survive formulation, storage, application, and field stress. Formulation needs to preserve microbial viability and compatibility among consortium members. Depending on the formulation, carrier materials may need to buffer desiccation, retain moisture, support gradual delivery, or reduce detrimental interactions among consortium members. Carrier systems also need to avoid favoring pathogenic agrobacteria or substantially disturbing resident soil organisms. Field establishment requires more than short-term survival of the inoculum. Relevant consortium members must persist in the target niche and retain the function for which they were selected [50].

Ecological establishment can be assessed at strain, community, and functional levels. Strain-specific tracking or culture recovery can show whether individual members survive after application, while community and functional measurements can determine whether the intended functions persist. In multi-strain formulations, storage and field conditions may alter strain ratios even when the total inoculum abundance remains high. Formulation should therefore preserve both member viability and functional balance.

### 6.3. Integration with Soil Health Practices

Soil amendments or management changes are most likely to help when they address a site-specific constraint. They need not accompany every microbial treatment. Organic amendments may be useful when low organic matter or poor microbial activity constrains antagonist establishment, whereas pH adjustment or irrigation management may be more relevant at other sites [41,42]. Compatibility with the consortium needs to be tested before combined application because an amendment that supports one member may alter strain ratios or provide additional resources to pathogenic agrobacteria.

Fertilizers, pesticides, and other routine inputs should be evaluated under the management regime in which the consortium will be used because they can alter consortium survival and suppressive activity. The relevant outcome is the maintenance of microbial functions associated with lower pathogen persistence and reduced infection; increases in total microbial biomass or diversity alone are insufficient.

### 6.4. Field-Level Constraints and Unresolved Challenges

Field heterogeneity is likely to be a major source of inconsistency in microbiome-based crown gall management [77]. Soil texture, pH, organic matter, moisture, host genotype, cropping history, climate, and resident microbial communities differ among nurseries and orchards [88]. A consortium that establishes in one soil may fail in another even when pathogen pressure is similar. This limits the generalizability of fixed formulations developed under a single set of greenhouse conditions [87]. Crown gall adds further variability because pathogenic agrobacterial populations differ in host association, Ti plasmid background, resource use, and sensitivity to individual antagonists. Wound timing and location also vary among propagation, grafting, pruning, and environmental injury. A consortium that performs well against one pathogen population or at one infection window may therefore be less effective in another production setting.

The composition and activity of a microbial formulation can also shift between production and field establishment. Storage, carrier composition, inoculum density, strain ratio, and repeated subculture may alter viability or functional expression before application. After application, some members may disappear while others dominate. As a result, the relative abundance and persistence of consortium members in the field may differ substantially from those in the original formulation. Spatial delivery is another practical problem because a soil-applied inoculant may remain abundant in bulk soil while failing to establish at root collars or newly wounded tissues where infection occurs.

Fertilizers, pesticides, irrigation schedules, pruning, transplantation, and organic amendments may either support or disrupt the introduced community. Field validation therefore needs to test compatibility with the production system itself, not only with the target pathogen. Cost, shelf life, quality control, regulatory requirements, and the feasibility of multi-season monitoring will ultimately determine whether a biologically effective consortium can be used outside experimental plots [83].

### 6.5. Long-Term Monitoring and Adaptive Management

Long-term monitoring is needed to determine whether field performance remains stable or changes over time after application. Baseline measurements before treatment provide the reference point, while repeated sampling can reveal pathogen rebound, loss of consortium members, changes in soil conditions, or delayed disease recurrence. Sampling need not cover every niche uniformly; priority can be given to the sites most closely related to the ecological target of the intervention.

Persistent pathogen pressure may indicate that application timing is poorly matched to infection risk or that the delivery strategy does not adequately reach the relevant niche. Loss of the core antagonist may point to formulation, carrier, or soil-compatibility problems. Persistence of a supporting consortium member without disease suppression would indicate that establishment alone is insufficient and could reflect loss of the intended interaction, inadequate core-antagonist performance, or a misidentified site-specific constraint. Non-target disturbance would likewise argue against the current formulation. These observations can guide changes in strain composition, formulation, application timing or frequency, and soil management practices.

### 6.6. Biosafety and Non-Target Soil Ecological Effects

Biosafety assessment is necessary for microbial consortium application in crown gall-affected soils. Candidate strains require screening for pathogenicity, antibiotic resistance traits, toxin production, undesirable plant effects, and potential risks to non-target organisms. Genome-based screening for virulence factors, mobile genetic elements, and antibiotic resistance genes can identify additional risks before field application. Key concerns include horizontal gene transfer, persistence beyond the intended niche, and unintended shifts in soil microbial community structure. Although locally adapted strains may be more compatible with resident communities than non-local inoculants, local origin alone does not guarantee safety [87].

Non-target assessment should focus on whether disease control is achieved at the cost of wider ecological disruption. Major disruption of beneficial microbial populations, nutrient-cycling functions, soil enzyme activity, or plant performance would weaken the case for long-term use even when gall incidence is reduced. Functional monitoring is also relevant because undesirable resistance, virulence, or mobile-element-associated traits may increase in the surrounding community, even when the introduced strains themselves pass genome-based safety screening.

Perennial systems require particular caution because introduced or reinforced microbial groups may interact with the soil ecosystem over multiple seasons. Long-term persistence may be desirable when it supports disease-suppressive functions, but it can raise ecological concerns if introduced microbial populations substantially alter the resident community structure or spread beyond the intended application niche. Biosafety assessment should continue throughout field development, particularly when consortium members are expected to persist across multiple seasons.

### 6.7. Application in Nursery and Perennial Production Systems

**Figure 3:**
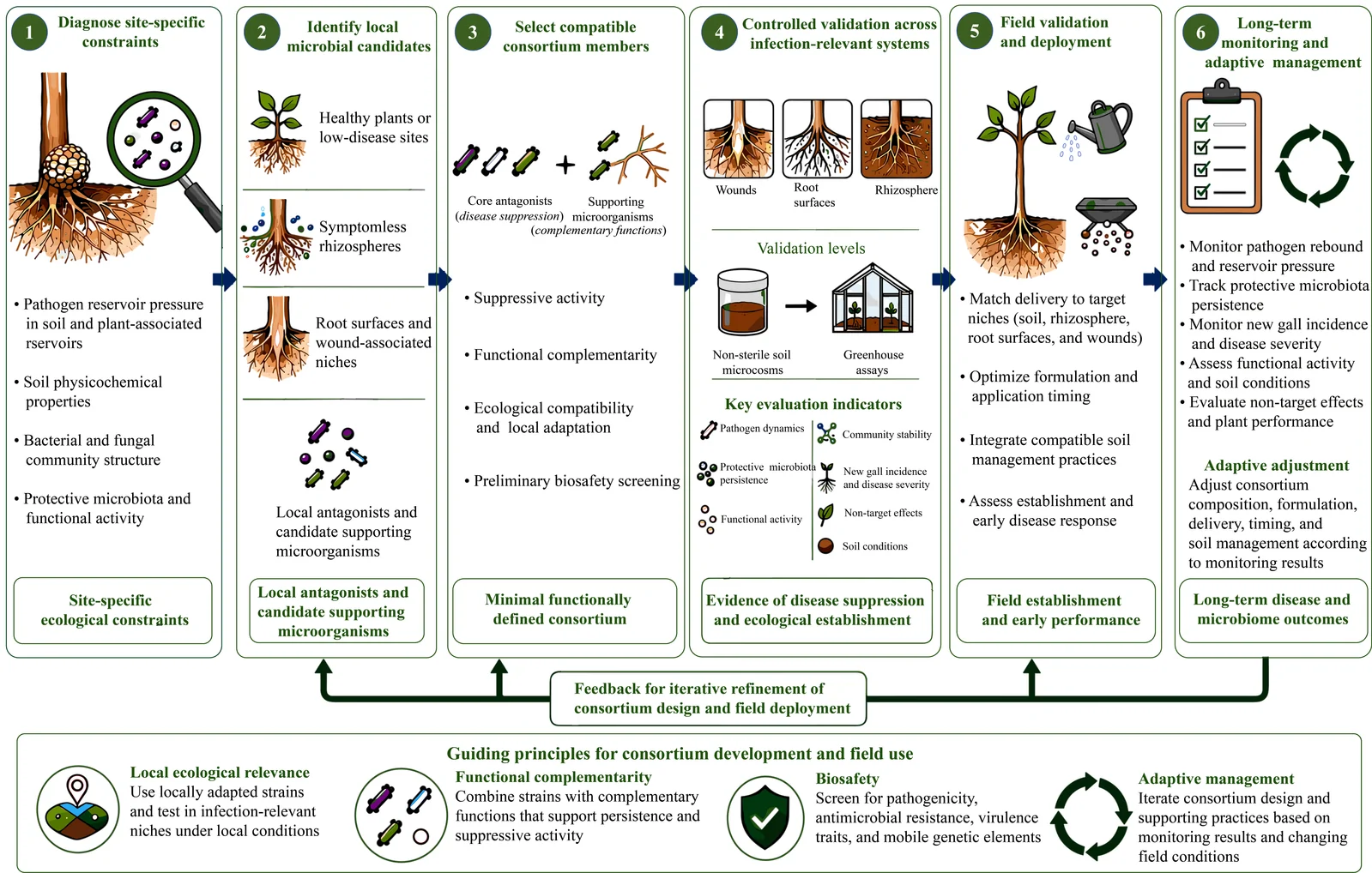
Proposed staged framework linking field implementation, long-term monitoring, and future research priorities for microbiome-based crown gall management. The six steps represent (1) diagnosis of site-specific constraints, (2) identification of locally adapted microbial candidates, (3) selection and assembly of compatible consortium members, (4) controlled validation across infection-relevant systems, (5) field validation and deployment, and (6) long-term monitoring and adaptive management. Evaluation integrates pathogen dynamics, persistence of protective microbiota, functional activity, soil conditions, new gall incidence and disease severity, microbial community stability, and non-target effects. Monitoring outcomes provide feedback for subsequent refinement of consortium composition, formulation, delivery, timing, and soil management.

Nurseries offer relatively defined intervention points because planting material, grafting, transplantation, and wound treatment can be managed before plants enter long-term production. Established perennial systems present a different problem because soil and root reservoirs are already present and infection windows recur over several seasons. Nursery applications can therefore emphasize prevention and the early occupation of wounds and root-associated niches, whereas perennial systems require sustained control of pathogen reservoirs and repeated monitoring of protective populations. Figure 3 integrates these translational steps with formulation, delivery, biosafety, and adaptive management.

## 7. Conclusions and Future Research Priorities

Crown gall recurrence is sustained by persistent agrobacterial reservoirs and repeated access to wounded tissues, but the surrounding microbiota are likely to influence how readily pathogenic populations expand from these reservoirs and recolonize infection sites. Sanitation, wound protection, and established biological control should remain the basis of management, with microbiome-based interventions providing a complementary option where protective populations fail to persist or recover under local soil conditions.

The key unresolved question is which microbial features are causally linked to reduced crown gall incidence. Compartment-resolved and longitudinal studies are needed to identify bacterial and fungal taxa, interactions, genes, and metabolites that consistently accompany low crown gall incidence and to test these candidates experimentally. Candidate consortia should remain minimal and functionally defined and should be evaluated across contrasting soils, hosts, pathogen populations, and seasons. Field assessment should combine new gall incidence with pathogen rebound, persistence of protective microorganisms, functional activity, soil conditions, and non-target effects. Figure 3 links the field implementation pathway described in Section 6 with these future research priorities, from mechanistic validation to adaptive field management. As climate-driven environmental variability increases and pressure grows to reduce reliance on broad-spectrum antimicrobial inputs, robust field evidence for these approaches will become increasingly important.

## Figures and Tables

**Table 1:** Major crown gall management approaches and their ecological scope and limitations.

Management Approach	Representative Example(s)	Main Target and Mechanism	Evidence/Application Status	Main Ecological Limitation	Reference(s)
Agronomic and preventive management	Clean planting material; quarantine; sanitation; tool disinfection; wound and stress management	Reduces pathogen introduction, movement, and access to fresh wounds before infection is established	First-line preventive management; most useful before long-term reservoirs are established	Cannot reliably remove established soil or plant-associated reservoirs; latent infection and repeated wounding remain	[13]
Chemical and wound-protection approaches	Disinfectants, bactericides or antibiotics where permitted; wound protectants	Locally lowers pathogen load or protects susceptible wound sites during high-risk stages	Useful for local or short-term risk reduction when timing and coverage are appropriate	Limited effect after stable host transformation; potential non-target effects, resistance selection, residues, and regulatory constraints	[18]
K84/K1026-type single-strain biocontrol	*A. radiobacter* K84; K1026	Targets susceptible to pathogenic agrobacteria during early infection; agrocin-mediated antagonism is central to the K84 system	Established preventive biocontrol for nursery and wound protection in susceptible pathogen populations	Strain specificity and context-dependent establishment; mainly preventive and does not address the wider soil reservoir	[19,20,21,22,23]
Other biological antagonists	Nonpathogenic *A. vitis* strains such as ARK-1; *B. velezensis* strains	Direct antagonism, interference with virulence-related processes, competition, biofilm-associated persistence, or plant-mediated protection	Evidence includes controlled experiments and selected field evaluations	Performance can vary with pathogen population, host, soil conditions, colonization, and formulation; field reproducibility remains uneven	[26,27,28,29,30,31,33,36,37]
Host-mediated approaches	Resistant rootstocks; altered root-exudate profiles	Changes pathogen recruitment and host susceptibility and may alter the root-associated chemical environment	Complementary or emerging strategy that acts before or during early colonization	Often host- or genotype-specific; effects may vary among rootstocks, soils, and production systems	[34,35]
Microbiome-supportive soil management	Compost or organic amendments; soil health practices; pH, irrigation, or nutrient management	Modifies the physicochemical environment and resident microbiome that shape competition, colonization resistance, and pathogen rebound	Supportive ecological management; strongest evidence is currently drawn from broader soil-borne disease systems	Strong site dependence; poorly matched amendments can also favor pathogen persistence or alter non-target functions	[38,39,40,41,42]
Proposed consortium-assisted management	Locally adapted core antagonists combined with functionally selected supporting microorganisms	Aims to improve persistence and suppressive function through microhabitat support, resource facilitation, stress tolerance, biofilm support, or functional complementation	Proposed approach; direct crown gall-specific validation remains limited	Consortium compatibility, strain ratios, formulation, niche-specific delivery, and stability across soils and seasons remain unresolved	[43,44,45,46,47,48,49,50]

**Table 2:** Microbial functions potentially supporting antagonist performance in crown gall-associated soil management.

Supporting Function	Mechanistic Contribution	Relevance to Crown Gall	Expected/Testable Outcome	Reference(s)
Soil microhabitat improvement	Modifies the local microenvironment, including pH/redox conditions, moisture retention, aggregation, or nutrient availability	May create local conditions that improve establishment of adapted protective microorganisms	Improved persistence and functional activity of the core antagonist or protective guilds	[41,42,54,71]
Nutrient facilitation	Provides or redistributes accessible carbon, nitrogen, amino acids, vitamins, or other growth-supporting metabolites	Can support protective taxa when local resources limit their establishment or activity	Higher or more stable activity of protective microorganisms without promoting pathogen rebound	[45,57,81]
Biofilm support	Promotes attachment, aggregation, matrix production, or stable colonization on roots, wounds, or soil particles	May improve persistence at infection-relevant interfaces where pathogenic agrobacteria encounter the host	More stable occupation of root surfaces, rhizosphere niches, or wound-associated sites	[80]
Iron and resource modulation	Changes siderophore-mediated iron competition or competition for other limiting resources	May reduce the resource advantage of pathogenic agrobacteria while favoring compatible protective populations	Lower pathogen reservoir pressure or weaker pathogen rebound	[53]
Stress buffering	Improves antagonist tolerance to oxidative, drought, salinity, pH, temperature, or other field-related stresses	Supports antagonist activity under variable soil and production conditions	More stable suppressive function across environmental fluctuations	[45,46,50]
Plant-mediated facilitation	Alters root-associated conditions, recruitment of protective microorganisms, or plant defense-related responses	May make the rhizosphere or wound environment less favorable to pathogen colonization	Reduced infection probability together with maintained plant performance	[31,32,72,73,74,75,76]
Functional complementation	Adds competitive, antimicrobial, anti-virulence, signaling-interference, or colonization functions that complement the core antagonist	Broadens suppression beyond one strain, one metabolite, or one infection niche	Greater functional breadth and lower risk of failure when one suppressive mechanism is weak	[45,46,81]
Community stabilization	Supports compatible interactions, functional redundancy, and recovery of protective guilds after disturbance	May prevent collapse of introduced or reinforced protective functions in non-sterile soils	Greater stability of disease-suppressive functions over time or after disturbance	[63,78,82]
Cross-kingdom facilitation	Provides complementary functions through bacterial–fungal interactions, such as substrate turnover, root association, spatial structuring, or stress buffering	May supply ecological functions that are weak or absent in bacterial-only consortia; direct crown gall evidence remains limited	Improved functional complementarity when fungal participation is supported by local evidence	[57,58,59,60,61]

Note: Evidence supporting these functions varies in strength and disease context. Crown gall-specific evidence remains limited, particularly for cross-kingdom interactions. Candidate consortium members should therefore be retained only when the proposed function is experimentally supported under relevant conditions.

**Table 3:** Indicators for evaluating microbiome rehabilitation in crown gall-affected soils.

Indicator Level	Core Measurement	Sampling Niche	Monitoring Window	Evidence Supporting Rehabilitation	Reference(s)
Disease phenotype	Incidence or severity of newly formed galls	Root collar and susceptible wound sites	Medium- to long-term	Sustained reduction in new gall incidence or severity across repeated assessments	[18,27,30]
Pathogen reservoir	Culture-based quantification with confirmation of pathogenicity or Ti plasmid carriage and/or quantitative PCR (qPCR) targeting Ti plasmid-associated markers	Bulk soil, rhizosphere, root surface, and wound-associated niches	Repeated time points	Lower pathogen reservoir pressure in infection-relevant niches	[12,13]
Pathogen dynamics	Temporal fluctuation and rebound of pathogen abundance	Soil, rhizosphere, roots, and propagation-associated niches	Long-term/across production cycles	Weaker rebound after disturbance or renewed production and sustained restriction of pathogen abundance over repeated measurements	[12]
Protective microbiota persistence	Abundance, recovery, or strain-specific tracking of core antagonists and resident protective guilds	Rhizosphere, root surface, and wound-associated niches	Repeated time points	Persistent establishment of protective microorganisms at sites relevant to infection	[23,24]
Community composition	Bacterial and fungal community composition, beta-diversity, and temporal stability	Bulk soil and rhizosphere	Repeated time points	Persistent treatment-associated shift or greater stability that accompanies disease suppression; increased diversity alone is insufficient	[51,86]
Network organization	Recurrent associations, candidate hub/connector taxa, network robustness, or recovery after disturbance	Bulk soil and rhizosphere	Multiple time points	Recurrent network features associated with low disease or improved recovery; network topology alone does not establish causality	[63,79]
Functional activity	Antimicrobial activity, siderophore-mediated competition, biofilm traits, anti-virulence activity, and relevant functional genes or metabolites	Rhizosphere and wound-associated niches	Medium- to long-term	Sustained suppressive activity that changes in parallel with pathogen reduction and disease outcome	[28,52,53,69]
Plant response	Defense-related or induced-resistance indicators together with plant growth	Roots, root collars, and susceptible wound tissues	Short- to medium-term	Defense-associated response without an evident growth penalty	[31,32]
Soil physicochemical context	pH, organic matter, moisture, nutrient status, and selected physical properties	Bulk soil and rhizosphere	Baseline plus repeated sampling	Conditions remain compatible with protective microbial activity and do not favor rapid pathogen rebound	[71]
Biosafety and non-target effects	Non-target microbial and plant responses, soil functional indicators, and screening for antimicrobial resistance genes, virulence genes, or mobile genetic elements where relevant	Multiple soil and plant-associated niches	Medium- to long-term	No major disruption of non-target microbial communities or soil functions and no undesirable enrichment of risk-associated traits	[87]
Integrated assessment	Concordance among disease, pathogen, protective microbiota, community, function, plant, and soil indicators	Multiple niches and production stages	Across seasons	Consistent improvement across several indicator levels and repeated sampling periods	[85]

Note: Evidence for microbiome rehabilitation should rely on concordant changes across multiple indicator levels rather than on any single measurement.

## Data Availability

No new data were created or analyzed in this study. Data sharing is not applicable to this article.

## Supplementary Materials

The following supporting information can be downloaded at: https://www.mdpi.com/article/10.3390/microorganisms14092089/s1, Table S1, Evidence levels for microbial mechanisms and microbiome-based management concepts relevant to crown gall.
