# Supplementary material for: Ecological Management of Crown Gall-Affected Soils: Pathogen Reservoirs, Disease-Suppressive Microbiomes, and Helper Consortia
Source: Microorganisms. 2026 Sep 18;14(9):2089. doi: 10.3390/microorganisms14092089 (PMC13609791; doi:10.3390/microorganisms14092089)
Supplement: Supplementary file 1 [file microorganisms-14-02089-s001.zip › microorganisms-4470080-supplementary.pdf]

Table S1. Comparison of major control strategies for long-term crown gall management

Control strategy	Main target	Time scale	Stability of control efficacy	Impact on the microbiome	Major limitations
Agronomic and preventive management	Pathogen introduction, transmission, and wound-associated infection	Short- to medium-term	Moderate; dependent on consistent implementation and clean planting material	Mostly indirect; may reduce pathogen entry but rarely rebuild suppressive functions	Difficult to eliminate established soil reservoirs, latent infections, or pathogen persistence in plant residues
Chemical, antibiotic, and wound-protection approaches	Local pathogen load and early wound infection	Short-term	Often limited and affected by environmental variation and application timing	Possible non-target disturbance to resident microorganisms	Resistance risk, environmental residues, regulatory restrictions, and limited effect on established galls
Single-strain biocontrol	Pathogens and early infection events	Short- to medium-term	Highly context-dependent; influenced by pathogen sensitivity, colonization, and soil conditions	Localized microbial regulation, usually without broad microbiome rehabilitation	Unstable colonization, narrow mechanisms, and limited resilience to environmental disturbance
Multi-strain biocontrol or synthetic microbial consortia	Soil, rhizosphere, root-surface, and wound-associated microecosystems	Medium- to long-term	Potentially improved but dependent on compatibility, colonization stability, and formulation quality	Potentially compatible with soil microbiome functions if non-target effects are limited	Complex design, formulation stability, field reproducibility, and ecological monitoring requirements
Soil microbiome rehabilitation-oriented management	Microbiome structure, ecological resistance, pathogen reservoirs, and disease-suppressive functions	Long-term	Potentially durable when supported by monitoring, adaptive management, and stable microbial functions	May improve microbial diversity, resilience, functional redundancy, and beneficial-to-pathogen balance	Requires standardized indicators, long-term monitoring, biosafety assessment, and site-specific optimization
